# Effects of the Multidimensional Treatment on Pain, Disability, and Sitting Posture in Patients with Low Back Pain: A Randomized Controlled Trial

**DOI:** 10.1155/2021/5581491

**Published:** 2021-06-30

**Authors:** Tae-Sung In, Jin-Hwa Jung, Kyoung-Sim Jung, Hwi-Young Cho

**Affiliations:** ^1^Department of Physical Therapy, Gimcheon University, 214 Daehak-ro, Gimcheon-si, Gyeongsangbuk-do 39528, Republic of Korea; ^2^Department of Occupational Therapy, Semyung University, 65 Semyung-ro, Jecheon-si, Chungcheongbuk-do 27136, Republic of Korea; ^3^Department of Physical Therapy, Gachon University, 191 Hambangmoe-ro, Yeonsu-gu, Incheon 21936, Republic of Korea

## Abstract

The purpose of this study was to investigate the effects of multidimensional approach model on the pain, disability, and sitting posture in patients with nonspecific low back pain (LBP). Sixty LBP patients were recruited and were randomly divided into two groups: multidimensional treatment (MT) group (*n* = 30) and unimodal treatment (UT) group (*n* = 30). All participants underwent 48 sessions of treatment (40 min/session, two sessions per day, 2 days per week) for 12 weeks. The MT group conducted a core stability exercise twice a day and additionally provided training on pain principles and management methods. The UT group only performed a core stability exercise twice a day. The visual analog scale (VAS) and Oswestry Disability index (ODI) were used to measure pain intensity and disability. Thoracolumbar kyphosis and lumbar lordosis in the sitting position were measured using a motion capture system. After training, the pain and disability in the MT group improved significantly greater than the UT group (*p* < 0.05). In the MT group, the pain relief effect persisted 3 months after the end of training. Thoracolumbar kyphosis and lumbar lordosis in the MT group were significantly improved compared to the UT group (*p* < 0.05). Thus, MT combined with core stability exercise may be used to improve the pain, disability, and sitting posture in patients with LBP.

## 1. Introduction

Low back pain (LBP) is one of the most common musculoskeletal disorders in the world, and about 90% of patients have nonspecific LBP with no obvious cause [1]. Lifestyle factors, such as sedentary jobs and obesity, have further increased the incidence of back pain [2]. Keegan suggested that decreased lordosis in the lumbar spine is the chief contributing factor in LBP caused by extensive sitting [3]. Depending on sitting posture, activities of various trunk muscles show different patterns [4]; in one study comparing sitting posture between patients with LBP and healthy subjects, LBP was associated with greater thoracic kyphosis [5, 6].

In LBP patients, pain causes muscle activation patterns to change, such as coactivation between agonists and antagonists [7]. However, Hodges demonstrated that these patterns can be retrained and controlled using therapeutic core exercise [8]. Core stability exercise is a training method that uses the motor learning principle to promote coordination of the deep trunk musculature. Recent studies have shown that it has various effects in patients with LBP [9–11]. This training method begins with low-level isometric contraction of core stabilizing muscles and progresses to performing functional tasks [9]. In a review, core stability exercise had more significant effects than general exercise with regard to improvements in pain and function in LBP patients [12].

Exercise improves the symptoms of LBP patients by improving muscle activity patterns. However, these effects are difficult to maintain after training is completed [13]. Therefore, to prevent the symptoms of LBP from worsening, clinicians should provide education on lifestyle habits that worsen the condition, including posture [14]. In a study that applied the multidimensional self-management program in LBP patients, pain, dysfunction, and mental health were significantly better than in those who received unimodal treatment. Also, this method may reduce the cost of treatment and follow-up management. Several previous studies have reported that the multidimensional program is more effective at reducing pain in LBP patients than unimodal treatments [15–17], but few studies have addressed the long-term effects of treatment. In addition, most previous studies have focused on the effects of training on pain and motor function, while little research has asked whether the training improves patients' usual posture.

This study aimed to investigate the effects of multidimensional treatment methods, including core stabilization exercise, patient education, and lifestyle guidance, on the pain, function, and sitting posture of LBP patients. In addition, we investigated the follow-up effect of multidimensional treatment on the change of pain in patients with LBP.

## 2. Subjects and Methods

### 2.1. Participants

**Figure 1:**
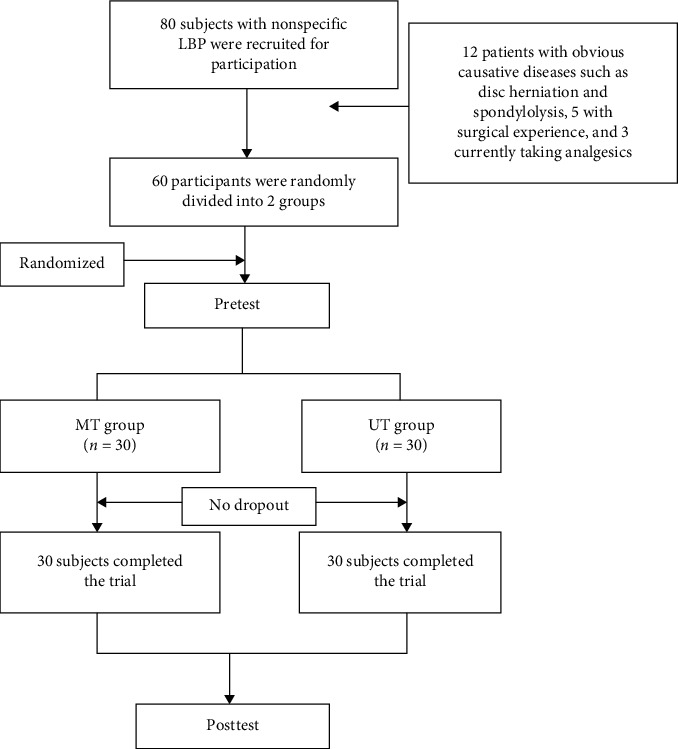
Flow diagram of participants through the study.

Sixty patients with LBP were recruited from J Hospital in the South Korea. All subjects had experienced nonspecific LBP for 3 months or longer without abnormal findings on radiographic examination, were aged between 18 and 65 years, and had a visual analog scale score of 3 or higher. The following exclusion criteria were applied: LBP due to a specific disease, history of spinal fractures, history of spinal surgery in the previous 2 years, inability to sit independently, inability to independently fill out a questionnaire, current LBP-related drug use, and recent participation in a similar exercise program. Of the 80 recruited, 12 patients with obvious causative diseases such as disc herniation and spondylolysis, 5 with surgical experience, and 3 currently taking analgesics were excluded (Figure 1).

Table 1 shows the common characteristics of the participants. Informed consent was voluntarily obtained from all subjects before participation, and the study was approved by the Institutional Review Board (IRB) of Gachon University (IRB No. 1044396-201910-HR-186-01). The trial was registered under trial registration no. https://clinicaltrials.gov/ct2/show/KCT0004982.

We used the G^*∗*^ power 3.1.9.4 software (Heinrich-Heine-University Düsseldorf, version 3.1.9.4, Düsseldorf, Germany) to calculate the sample size. In the present study, the mean power was set at 0.77 and the alpha error at 0.05. Also, the effect size was set to 0.71 based on the pilot study (12 subjects). The analysis of G*∗* power software shows that at least 30 participants should make an acceptable group sample size for each group; thus 60 participants were recruited in the study.

### 2.2. Protocol

The outcomes were evaluated at the baseline and at one day after the last intervention session by three well-trained physical therapists who were not informed about the participants and purpose of the study. In addition, pain, which is the primary outcome in this study, was measured 3 months after the last session to measure the follow-up effect of the intervention. 60 patients were randomly allocated to the multidimensional treatment (MT) group (*n* = 30) and the unimodal treatment (UT) group (*n* = 30) using a selection envelope. A person who was otherwise uninvolved in the study selected a number (either 1 or 2) from a sealed envelope to ensure unbiased randomization.

The treatment consisted of a total of 48 sessions (40 min/session, two sessions per day, 2 days per week for 12 weeks). Interventions were applied twice a day, and training was performed on subjects in the morning and evening, respectively. In the MT group, participants performed one of the two daily core stability exercises under the supervision of therapist, while they performed the other themselves. Extensive education on pain principles and management methods was also conducted. In the UT group, guidance on self-core stability exercises, including stretching and walking, was provided by the same therapist and during the same time period as in the MT group; self-administration was then conducted twice a day without therapist supervision.

### 2.3. Intervention

In this study, MT treatment can be defined as training on pain management and lifestyle, including the supervision of the therapist for core stability exercise and exercise. The MT group performed 40 minutes of core exercise per session, over a total of 48 sessions for 12 weeks. Intervention was applied by modifying exercise method used in a previous study [18]. Training started with low-intensity isometric contraction of the core muscles that stabilize the trunk; the intensity was then gradually increased by performing functional tasks. The step-by-step composition of the 48 exercise sessions was as follows:

Phase 1 (8 sessions): independent isometric contraction of transversus-abdominis (trA) and multifidus
Phase 2 (12 sessions): cocontraction and functional tasks of the deep trunk muscles
  - Alternately lifting arms or legs in multiple positions (supine, quadruped, sitting, standing)
  - Segmental movement of thoracic spine
  - Bridging exercise
  - Cycling in a supine position
Phase 3 (12 sessions): functional task with load
  - Exercises in phase 2 were performed with an external load applied to wrist and ankle
Phase 4 (16 sessions): functional task with an unstable surface
  - Lifting both arms or legs while standing on a balance pad
  - Alternately raising arms and legs in a four-legged posture with chest rested on balance ball
  - Alternately raising arms and legs while sitting on balance ball
  - Side bending the trunk while sitting on balance ball

Before and after exercise, 5 minutes of warm-up and cool-down were provided. Subjects were instructed to maintain a neutral lumbar spine position while performing all functional movements. One session a day was conducted under the supervision of physical therapist with more than 5 years of experience, while the other session was performed by patients themselves. In addition, in the MT group, in addition to core training, additional education was conducted on (1) anatomy and kinesiology of the spine, including superficial and deep muscles of the trunk, (2) trunk muscle activity and muscle fatigue according to posture, (3) problems such as pain that can occur when the balance of trunk muscles is broken, (4) how to sit properly, and (5) a lifestyle or treatment method that can reduce pain. Subjects were instructed to perform trunk extension exercises (in sitting and standing positions according to the McKenzie method) and walk for 2-3 minutes every hour to reduce fatigue due to continuous muscle contraction of postural control muscles when sitting for a long time. In addition, patients were educated in detail about correct posture habits in daily life when sitting, standing, sleeping, carrying out self-hygiene (hair washing, face washing, and tooth brushing), and carrying things. On days of severe pain, they were instructed to lie down and take a break, and they were trained on how to apply an ice pack or hot pack, depending on whether they felt a sense of heat. On the other hand, on days where the pain is not severe, a method of releasing tight muscles using a foam roller or massage ball was also guided. Education was conducted for 4 hours in 2 sessions before the start of training.

The UT group was guided by the same therapist as the MT group on a 40-minute core stability exercise program that included stretching and walking to be performed by patients unsupervised.

### 2.4. Outcome Measurements

The VAS was used to measure the degree of pain in the subject. In order to make the timing of measurement of pain and factors that cause pain as similar as possible between subjects, the researchers asked the subjects the degree of pain they felt when they performed walking movements and other most uncomfortable movements for a week. For the VAS, a 10 cm long straight line marked with a score from 0 to 10 was used. Subjects were instructed to indicate the intensity of pain they felt on a straight line [19]. The test-retest reliability of VAS is *r* = 0.96 [20].

The Oswestry Disability Index (ODI) is a questionnaire-type measurement tool designed to be completed by patients to evaluate their functional disability arising from LBP. The scores therefore range from 0 (no disability) to 100 (maximum disability). In the ODI score distribution, 0 to 20 points indicate minimal dysfunction, 20 to 40 points indicate moderate dysfunction, and 40 to 60 points indicate severe dysfunction [21]. The test-retest reliability of the ODI Korean-version is very high (ICC = 0.9167) [22].

**Figure 2:**
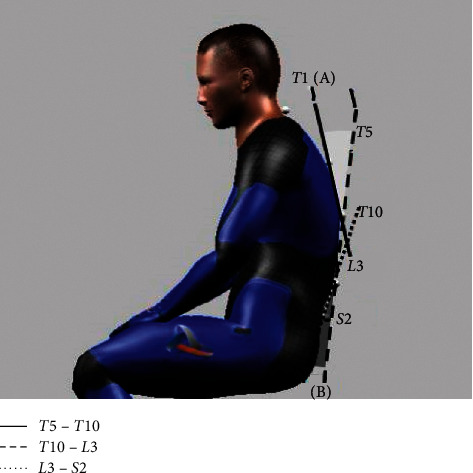
Measurement of thoracolumbar kyphosis and lumbar lordosis through 3D motion analysis system. (A) Thoracolumbar kyphosis angle and (B) lumbar lordosis angle.

Thoracolumbar kyphosis (TK) and lumbar lordosis (LL) were measured and recorded using a motion capture system consisting of 10 infrared cameras (Raptor-E; MotionAnalysis Inc., CA, USA). The camera was fixed on a tripod on horizontal ground 2 m away from the subject. Sampling frequency was 30 Hz, and markers were attached to the spinous processes of first, fifth, and tenth thoracic vertebrae, third lumbar vertebrae, and second sacral vertebrae. Kinematic data were analyzed using a video-motion analysis software called ORTHOTRAK (MotionAnalysis Inc., CA, USA). Mean error for marker position value of this motion analysis system is ±0.5 mm. Thoracolumbar kyphosis angle was measured as the angle between a line connecting the spinous processes of fifth and tenth thoracic vertebrae and a line connecting the spinous processes of tenth thoracic and third lumbar vertebrae. Lumbar lordosis angle was measured as the angle between a line connecting the spinous processes of tenth thoracic and third lumbar vertebrae and a line connecting the spinous processes of the third lumbar and second sacral vertebrae (Figure 2) [23]. The method of measuring thoracic and lumbar spinal curvatures with motion capture system is highly reliable with ICCs = 0.980 [24]. The measurements were repeated three times, and the average value was used for analysis.

### 2.5. Data Analysis

SPSS 21.0 (IBM, Armonk, NY, USA) was used for statistical analysis. The normality of variables was assessed using the Shapiro–Wilk test. The independent *t*-test for continuous variables and the chi-square test for categorical variables were used to compare the subjects' general characteristics between the MT and UT groups. The two-way repeated-measures ANOVA was conducted to compare pre- and posttest time with respect to the effect of multidimensional treatment (intervention) on pain intensity, disability, thoracolumbar kyphosis, and lumbar lordosis angle. The paired *t*-test was used as a simple effects analysis in each group when there was a significant interaction between the intervention and the time. The effect size was analyzed using Cohen's *d* and *f* values. In addition, we used the Pearson correlation coefficients analysis to analyze the correlation between pain (VAS), disability (ODI), and sitting position (TK and LL). The differences between the two groups were compared with the independent sample *t*-tests. The level of statistical significance was set at 0.05.

## 3. Results

Table 1 shows the characteristics of the participants in each group. There was no significant difference in any of the characteristics of participants. None of the subjects in this study complained of other chronic pain disorders such as migraine, fibromyalgia, and temporomandibular disorder.

**Figure 3:**
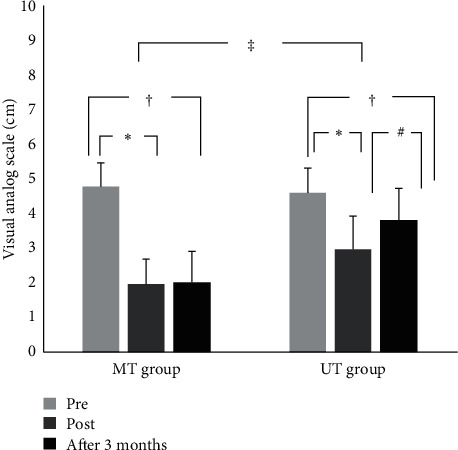
Change of VAS score after training. *∗*Significant difference between pre and post (*p* < 0.05). ^#^Significant difference between post and after 3 months (*p* < 0.05). ^†^Significant difference between pre and after 3 months (*p* < 0.05). ^‡^Significant difference between MT and UT group (*p* < 0.05).

A significant interaction between intervention and time on VAS was found (*F* (2, 116) = 39.241, *p* < 0.001). VAS significantly decreased between pre, post, and after 3 months in both the MT (4.86 ± 0.68 vs. 1.99 ± 0.73 vs. 2.02 ± 0.60, *F* (2, 58) = 160.042, *p* < 0.001, Cohen's *f* = 2.45) and the UT group (4.65 ± 0.72 vs. 3.01 ± 0.97 vs. 3.87 ± 0.93, *F* (2, 58) = 44.035, *p* < 0.001, Cohen's *f* = 0.93) (Figure 3). After training, the MT group showed more improvement in pain than the UT group, and even after 3 months after training (*t* = −5.304, *p*=0.001), there was a significant difference between groups in the degree of pain reduction (*t* = −4.842, *p*=0.001).

**Figure 4:**
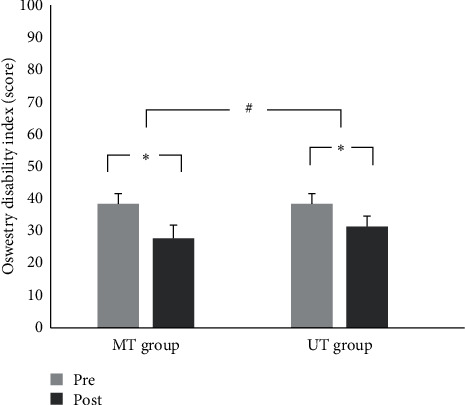
Change of ODI score after training. ^*∗*^Significant difference between pre and post (*p* < 0.05). ^#^Significant difference between MT and UT group (*p* < 0.05).

There was a significant interaction between intervention and time on ODI score (*F* (1, 58) = 17.191, *p* < 0.001). A simple effects analysis for intervention showed that there were significant decreases on ODI score in both the MT group (38.50 ± 3.79 vs. 28.13 ± 4.45, *p* < 0.001, Cohen's *d* = 2.50) and the UT group (37.80 ± 4.11 vs. 31.80 ± 3.40, *p* < 0.001, Cohen's *d* = 1.59) (Figure 4). After training, ODI was more significantly decreased in the MT group than in the UT group (*t* = −5.304, *p*=0.001).

**Figure 5:**
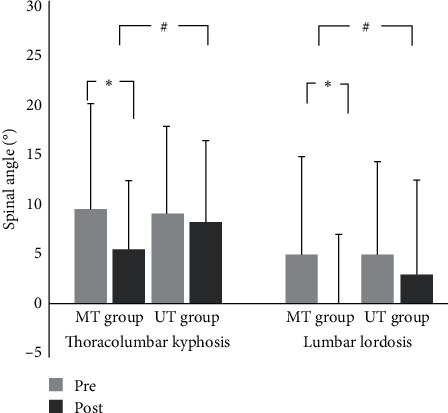
Change of spinal angle after training. ^*∗*^Significant difference between pre and post (*p* < 0.05). ^#^Significant difference between MT and UT group (*p* < 0.05).

There was a significant interaction between intervention and time on TK (*F* (1, 58) = 9.434, *p*=0.003) and LL (*F* (1, 58) = 8.270, *p*=0.006). A simple effect analysis for intervention showed that there were significant decreases on TK and LL in both the MT group (TK: 9.68 ± 10.51 vs. 5.54 ± 6.89, *p* < 0.001, Cohen's *d* = 0.47; LL: 5.01 ± 9.85 vs. 0.05 ± 7.02, *p* < 0.001, Cohen's *d* = 0.58) and the UT group (TK: 9.14 ± 8.76 vs. 8.31 ± 8.17, *p* < 0.001, Cohen's *d* = 0.10; LL; 4.85 ± 9.51 vs. 3.04 ± 9.47, *p* < 0.001, Cohen's *d* = 0.19) (Figure 5). The training caused a more significant decrease in TK and LL in the MT group than in the UT group (TK: *t* = −3.011, *p* < 0.005; LL: *t* = −2.883, *p* < 0.005, respectively).

Also, there were moderate correlations between VAS and ODI (*r* = 0.511, *p* < 0.001), VAS and TK (*r* = 0.531, *p* < 0.001), VAS and LL (*r* = 0.500, *p* < 0.001), ODI and TK (*r* = 0.561, *p* < 0.001), and ODI and LL (*r* = 0.388, *p* < 0.001) (Table 2).

## 4. Discussion

This study demonstrated the effect of a multidimensional approach model including core stabilization exercise and education on pain and function in LBP patients. Both groups had significant improvements in pain and disability after training. The stability of lumbar spine requires a harmonious interaction between passive system, active system, and neural system [25]. It is controlled by deep abdominal muscles such as multifidus and trA [26]. Several studies reported that, in LBP patients, multifidus shows atrophy or a decrease in cross-sectional area [27–30] and that these findings are associated with LBP recurrence [28, 31]. A previous study, performing core stability exercise in chronic LBP patients, showed significant increases in cross-sectional area of multifidus after intervention. Also, they reported that this exercise reduced spasm and mechanical irritation of lumbar region, thereby restoring the function of weakened spine and pelvic muscles in LBP patients [32]. In another study, muscle activity of trA increased after core stability exercise in healthy subjects. Such a change likely prevents back pain by reducing loading and excessive mobility in the spine when external loads are added [33].

Hodges et al. suggested that recovery of trunk muscle coordination via core stability exercises can improve trunk control ability [8]. Another study also showed that core stability exercise improved pain and dysfunction, which was related to the quality of trunk movement [34]. Similar to these findings, in our study, the MT group improved trunk stability with recovery of the motor control pattern of the trunk muscles after performing core stabilization exercise. Also, subjects showed improvement in pain and function. Interestingly, both groups performed core exercise for 12 weeks, but the MT group showed greater improvement in pain and disability than the UT group. This difference is thought to be due to the presence or absence of supervision by therapist. Although core exercise is helpful in treating LBP patients, this result suggests that it is more effective when expert supervision is involved to ensure more accurate performance. In addition, the MT group was educated on self-management methods (muscle relaxation using hot pack, massage ball, or foam roller) to reduce pain. It is assumed that this may have caused the MT group to have positive effective outcome compared to the UT group.

Mechanical stress increases in the lumbar spine when lumbar lordosis disappears in a sitting position [35]. Furthermore, sitting in improper posture over a long period increases lumbar pain and discomfort [36, 37]. Thus, we applied a multidimensional approach and proved that thoracolumbar kyphosis angle and the lumbar lordosis angle in a sitting position were significantly improved in the MT group compared to the UT group. Muscle activation varies depending on sitting posture [38], and a slumped posture with reduced lumbar lordosis causes chronic muscle degeneration, which habitually decreases muscle activity [39]. In the study, we reasoned that sitting posture was significantly better in the MT group than in the UT group because trunk muscle function was recovered after core stabilization and because the treatment method caused the patients to pay attention to proper posture habits. Furthermore, participants in the MT group were educated on anatomy and kinesiology of the spine, muscle imbalance according to postural change, and problems such as pain that may occur due to muscle imbalance and self-management methods to reduce pain. This seems to have motivated them towards correct posture habits in daily life.

Since this multidisciplinary program for LBP requires experts in various fields, it is not suitable for patient self-management in daily life [2], and the long-term pain reduction effect is reportedly poor [15–17]. Wippert et al. [2] showed significant improvements in pain, dysfunction, and mental health after applying a multidimensional self-management program that included psychological education for LBP patients. They also asserted that such programs could successfully be performed in everyday life, as the treatment effect persisted 6 months after training was completed, that strengthening the treatment component would increase the long-term sustainability of multidimensional therapy for a wide range of patients at various stages of LBP, and that the technique could also be applied to prevention. Thus, in the study, the MT group was educated so that they could continuously manage themselves in their daily lives, and pain intensity was evaluated 3 months after the end of training. The results showed that the subjects in the MT group reported similar pain intensity as immediately after the end of training and that the intensity was significantly lower than in the UT group, perhaps because the multidimensional intervention allowed the patients to manage themselves, because the patients' usual posture was improved through education and exercise, or because the factors that cause pain in daily life were greatly reduced. We confirmed that there was a moderate correlation between the amount of change in TK and LL, and the amount of change in pain and disability through Pearson correlation analysis. In a study on the posture analysis of patients with LBP, the subjects took a slumped posture before the onset of LBP, and the change in posture of patients with LBP was not due to a reflex response to pain, but due to a decrease in postural control ability [40]. In addition, Keegan reported that the most important factor in the onset of low back pain when sitting for a long time was a decrease in LL [3]. Therefore, it is believed that improved posture may be an important factor that may affect the reduction or recurrence of low back pain.

This study investigated the effect of a multidimensional treatment method that included core stabilization exercise and education on pain, function, and sitting posture in LBP patients. The results revealed that the MT group showed significantly better outcomes than the UT group in all outcomes and that pain reduction effect was maintained even at 3-month follow-up.

However, this study has some limitations. First, the measurement time of the posture is short, so it is difficult to say that it reflects all of the usual posture, and the long-term effect of training was not confirmed due to a relatively short follow-up period. Finally, since we did not include a natural history group in this study, there may be biases in the interpretation of the results. Thus, additional research is needed to confirm the difference in posture according to the presence or absence of education in order to more clearly confirm the effect of the interaction between education and training. In future studies, a variety of multidimensional treatment models should be developed to treat patients with LBP, to confirm the treatment effect in a long-term follow-up study lasting 12 months, and to measure different variables.

## 5. Conclusion

The results of this study suggest that multidimensional treatment including lifestyle changes may be helpful to nonspecific low back pain patients. However, the long-term effect of this study is unclear because follow-up observation after 6 months is not performed. Therefore, it is necessary to confirm the effect of multidimensional treatment through additional research.

## Figures and Tables

**Table 1:** Common and clinical characteristics of the subjects (*N* = 60).

Variables	MT group (*n* = 30)	UT group (*n* = 30)	*p*
Sex (male/female)	20/10	18/12	0.592^b^
Age (years)	41.13 ± 11.49^a^	40.63 ± 11.30	0.808^c^
Height (cm)	167.07 ± 7.63	166.97 ± 9.40	0.964^c^
Weight (kg)	65.83 ± 11.61	62.33 ± 11.06	0.237^c^
Duration of LBP (months)	11.50 ± 4.20	12.73 ± 5.98	0.359^c^

^a^Mean ± standard deviation, ^b^chi-square test, and ^c^independent *t*-test. LBP; low back pain.

**Table 2:** Correlation between pain, disability, and sitting posture.

	Pearson correlation coefficient	*p*
VAS vs. ODI	0.511	<0.001
VAS vs. TK	0.531	<0.001
VAS vs. LL	0.500	<0.001
ODI vs. TK	0.561	<0.001
ODI vs LL	0.388	<0.001

VAS, visual analog scale; ODI, Oswestry Disability Index; TK, thoracic kyphosis; LL, lumbar lordosis.

## Data Availability

The data used to support the findings of this study are available from the corresponding author upon request.
